# Lead Exposures in U.S. Children, 2008: Implications for Prevention

**DOI:** 10.1289/ehp.11241

**Published:** 2008-05-19

**Authors:** Ronnie Levin, Mary Jean Brown, Michael E. Kashtock, David E. Jacobs, Elizabeth A. Whelan, Joanne Rodman, Michael R. Schock, Alma Padilla, Thomas Sinks

**Affiliations:** 1 U.S. Environmental Protection Agency, Boston, Massachusetts, USA; 2 Centers for Disease Control and Prevention, Atlanta, Georgia, USA; 3 Food and Drug Administration, Washington, DC, USA; 4 Department of Housing and Urban Development, Washington, DC, USA; 5 National Institute for Occupational Safety and Health, Cincinnati, Ohio, USA; 6 U.S. Environmental Protection Agency, Washington, DC, USA; 7 U.S. Environmental Protection Agency, Cincinnati, Ohio, USA

**Keywords:** children’s health, environmental health, lead poisoning, primary prevention

## Abstract

**Objective:**

We reviewed the sources of lead in the environments of U.S. children, contributions to children’s blood lead levels, source elimination and control efforts, and existing federal authorities. Our context is the U.S. public health goal to eliminate pediatric elevated blood lead levels (EBLs) by 2010.

**Data sources:**

National, state, and local exposure assessments over the past half century have identified risk factors for EBLs among U.S. children, including age, race, income, age and location of housing, parental occupation, and season.

**Data extraction and synthesis:**

Recent national policies have greatly reduced lead exposure among U.S. children, but even very low exposure levels compromise children’s later intellectual development and lifetime achievement. No threshold for these effects has been demonstrated. Although lead paint and dust may still account for up to 70% of EBLs in U.S. children, the U.S. Centers for Disease Control and Prevention estimates that ≥30% of current EBLs do not have an immediate lead paint source, and numerous studies indicate that lead exposures result from multiple sources. EBLs and even deaths have been associated with inadequately controlled sources including ethnic remedies and goods, consumer products, and food-related items such as ceramics. Lead in public drinking water and in older urban centers remain exposure sources in many areas.

**Conclusions:**

Achieving the 2010 goal requires maintaining current efforts, especially programs addressing lead paint, while developing interventions that prevent exposure before children are poisoned. It also requires active collaboration across all levels of government to identify and control all potential sources of lead exposure, as well as primary prevention.

Some recent tragedies have evinced a more complicated risk pattern for pediatric lead exposures in the United States than had previously been considered:

- 21 April 2000, New Hampshire: A 2-year-old Sudanese refugee died from exposure to lead paint, the first U.S. child known to die from lead poisoning in 10 years [Centers for Disease Control and Prevention (CDC) 2005a].
- July 2002, New York City: A 1-year-old’s elevated blood lead level was traced to ceramic dinnerware without visible signs of wear (CDC 2004a).
- 23 July 2003, Massachusetts: A lead-coated copper wall and roof were identified in a child’s condominium where dust lead levels were 224,377 μg/ft^2^ (Brown MJ, unpublished memo to the Consumer Product Safety Commission, 2004).
- 2004, Oregon: A child was hospitalized after ingesting a necklace made with lead, resulting in voluntary recall of 150 million pieces of children’s jewelry (CDC 2004b).
- 23 March 2006: Minnesota: A 4-year-old died from lead poisoning after swallowing a charm with 99% lead content received with a purchase of shoes (CDC 2006).

The implications of these and similar events drove members of core federal agencies to jointly construct a more complete picture of potential lead exposures than had previously been compiled.

## Introduction

**Figure 1:**
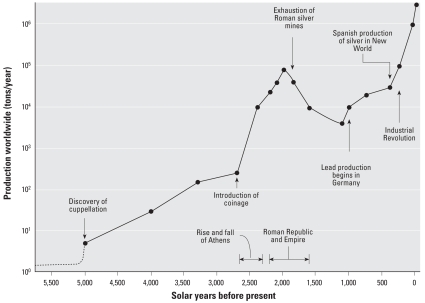
Increases in lead production and corresponding increases in lead emissions. Data from Davidson and Rabinowitz (1992) and U.S. EPA (1986).

Lead is corrosion-resistant, dense, ductile, and malleable and has been used since at least 3500 BCE. Atmospheric lead levels increased more than six orders of magnitude over the past six millennia accompanying population and economic growth (Figure 1) (Davidson and Rabinowitz 1992). Blood lead levels (BLLs) of U.S. children rose sharply between 1900 and 1975 as increased lead emissions caused widespread contamination. Changes in federal laws have reversed this trend, including eliminating leaded gasoline from on-road vehicles, banning the sale of leaded house paint, and prohibiting lead solder in public water systems, plumbing components, and food and drink cans. The sharp reduction in children’s BLLs between 1976 and 1989 demonstrates that these policies have been effective (Mahaffey et al. 1982; Pirkle et al. 1998). However, children continue to be exposed to lead. In 1999–2002, an estimated 310,000 (1.6%) U.S. children had BLLs ≥10 μg/dL, and 1.4 million had BLLs of 5–9 μg/dL (almost 14%) (CDC 2005b).

The adverse health effects of lead—including death, insanity, nervous system damage, and sterility—have been reported since the second century BCE (Major 1945). Even low lead exposure affects children’s intellectual development and lifetime achievement. Since the 1980s, studies have linked BLLs < 10 μg/dL in children 1–5 years of age with decreased IQ and cognition, with demonstrated effects evident at about 2 μg/dL (Jusko et al. 2008). No threshold for effects has been demonstrated.

In 2000, the United States adopted the goal of reducing all exposures to lead and eliminating elevated blood lead levels (EBLs; BLLs ≥10 μg/dL) in children by 2010 (Department of Health and Human Services 2000). However, projections of future decreases in the number of children with EBLs (Jacobs et al. 2002) assume a funding schedule that is not fully actualized. The nation’s goal to eliminate childhood BLLs > 25 μg/dL by 2000 was not met (Jacobs and Nevin 2006). The 2010 goal may fall short without augmented investment.

Screening children for lead and abating lead paint hazards in homes of children with EBLs must continue. But given ubiquitous lead contamination, merely reducing hazards in residences of children identified with EBLs will not suffice. Childhood lead poisoning prevention programs (CLPPPs) must consider current and past uses of lead as well as behaviors that leave specific populations vulnerable to excessive lead exposures. To be effective, CLPPPs must shift to primary prevention.

## Sources of Lead Exposure

Deteriorating lead paint and contaminated dust and soil are the primary, but not the only, causes of EBLs among U.S. children. Lead is used in thousands of applications, all of which constitute potential exposure sources [U.S. Environmental Protection Agency (EPA) 2006a]. Recent data indicate that ≥30% of children with EBLs do not have an immediate lead paint hazard. For example, in 2004 in Arizona, soil was the most common identified proximate exposure source, accounting for about 24% of pediatric EBL cases, followed by paint (17%), folk remedies and pottery (17%), dust (15%), and miscellaneous other sources (19%). In 8% of cases, no lead source was identified (Arizona Department of Health Services 2005).

Nonpaint lead exposure sources are insufficiently characterized, and their importance is often underestimated. When a child with an EBL is reported, investigators look for lead paint in places where s/he spends time, exploring alternative lead exposure sources only when no paint hazards are found. Thus, for some children, significant nonpaint sources may be missed. Evidence also suggests that for children with BLLs < 10 μg/dL, no single exposure source predominates (Bernard and McGeehin 2003).

### Lead in the environment

The United States is the third largest lead producer, producing about 450,000 tons in 2003 (U.S. Geological Service 2004). In 2003, the United States consumed about 1.5 million tons of lead (Commodity Research Bureau 2006). Facilities using lead can raise exposures for adjacent populations. Not all sources are obvious, and many users are exempt from reporting. In Massachusetts in 2003, for instance, 252 facilities used nearly 9.3 million pounds of lead, with the largest releases reported by municipal waste combustors (Table 1).

#### Air

During the 20th century, leaded gasoline was the predominant source of airborne lead. Today, industrial emissions predominate. In 2001, the U.S. Environmental Protection Agency (EPA) reported that industrial emissions accounted for 78% of air lead, fuel consumption accounted for 10%, and the transportation sector accounted for 12% (U.S. EPA 2007a). In 2004, four waste treatment plants were among the 20 largest dischargers of lead submitting data to the Toxics Release Inventory (TRI) of the U.S. EPA (U.S. EPA 2007d).

**Figure 2:**
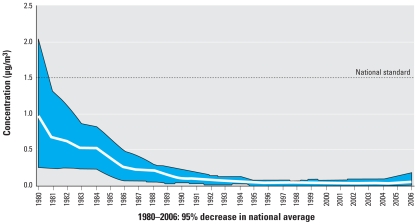
Maximum quarterly mean air lead concentrations, 1980–2006, showing 95% decrease 1980–2003 and slight increase 2004–2006; national trend based on 15 sites. Reprinted from U.S. EPA (2007a).

After declining for > 25 years, U.S. air lead levels rose in 2004–2006 (Figure 2) (U.S. EPA 2007a). The highest air concentrations of lead are found near smelters and battery manufacturers. At present, these are the only violations of the national air lead standards (U.S. EPA 2007a). However, national air lead emission data cannot accurately portray local lead emissions or their risk for proximate populations. Exposure modeling at the U.S. EPA indicates that for the 20 highest air emitters, local emissions are significantly related to local BLLs (U.S. EPA 2007b).

Not all sources of lead are listed in the U.S. EPA TRI. Municipal incinerators, small operations such as auto repair shops, off-road vehicles including NASCAR, and propeller aircraft using aviation gasoline (avgas) are exempt from reporting, fall below reporting quantities, or choose not to report; nonetheless, they can contaminate surrounding communities. For example, at one airport where many airplanes used avgas, average and maximum air lead levels were 0.030 and 0.302 μg/m^3^, respectively, versus background levels of 0.007 and 0.018 μg/m^3^ (Environment Canada 2000). Another study showed that even at an airport with few planes using avgas, air lead levels were higher downwind than upwind (Illinois Environmental Protection Agency 2002).

Demolition of old buildings contributes to local air lead levels and can increase BLLs in children (Farfel et al 2003; Rabito et al 2007).

#### Soil

Lead binds tightly to soils, and eight decades of leaded gasoline combustion and past industrial emissions have left a legacy entrained in soil. Peeling lead paint on residences also contaminates soil, especially in distressed neighborhoods. Because of higher traffic levels and denser housing, the soil in urban areas can average 800–1,200 μg/g (Duggan and Inskip 1985; Lanphear 1998a). Soil from play areas has a larger impact on children’s BLLs than soil from other areas (Lanphear et al. 1998b; Mielke and Reagan 1998). Lead tire weights that fall off are quickly abraded and ground into tiny pieces by traffic, resulting in high dust-loading rates, especially in urban areas (Root 2000). Lead exposure also occurs through produce grown in contaminated soil (Finster et al. 2004).

Children living near mining and smelting sites are at risk for EBLs (Maisonet et al. 1997; Murgueytio et al. 1996; Swarup et al. 2005). Studies find effects even 20 years after smelter closing (Diaz-Barriga et al. 1997).

Historical research to uncover past commercial activities can identify current sources of exposure (Eckel et al. 2001). For instance, a Washington State study (Wolz et al. 2003) found that homes near locations where lead arsenate was used as a pesticide between 1905 and 1947 had significantly higher soil and indoor dust levels.

Elevated soil lead levels are found at more than two thirds of Superfund sites in all 50 states [Agency for Toxic Substances and Disease Registry (ATSDR) 2005]. Lead is the chemical most frequently released from uncontrolled hazardous waste sites; in 1997, the ATSDR identified lead contamination in 59% of the sites monitored (ATSDR 2005). Numerous historical mining and smelting districts are now Superfund sites (Spalinger et al. 2007).

BLLs can rise 1–5 μg/dL for every 1,000-ppm increase in soil lead (U.S. EPA 2006a).

#### Dust

Dusts are composed of fine particles of soil, paint, and industrial or automotive emissions. They accumulate on exposed surfaces and are trapped in clothing and carpet fibers. Ingesting dust particles is the typical route of lead exposure for children (U.S. EPA 2006a). Dust is absorbed more readily than either paint or soil; house dust levels best predict children’s BLLs (Lanphear et al. 1998c). Consequently, regulations for lead abatement and remediation have included dust clearance standards that quantify lead concentrations [Department of Housing and Urban Development (HUD) 1999; U.S. EPA 2006c].

BLLs can rise 1–5 μg/dL for every 1,000-ppm increase in dust lead (U.S. EPA 2006a).

### Lead in the diet

The sources of lead in food may be natural or anthropogenic, and contamination can occur at any point in processing through contact with metal implements, solder, pigments, glazes, or packaging. Lead also enters food from drinking water, serving utensils, and household dust. Dietary exposures in the United States are 1–4 μg lead per day [U.S. Food and Drug Administration (FDA) 2006a], and have remained fairly constant during the past decade. Foreign manufacturers who fail to meet U.S. standards can produce contaminated food.

#### Breast milk

Lead in breast milk is related to current maternal exposures and to past exposures mobilized from lead stored in bones (Chien et al. 2006). Even low levels of lead in breast milk strongly influence an infant’s BLL (Ettinger et al. 2006). Calcium supplementation can reduce lead in breast milk. In a randomized trial, calcium supplements lowered BLLs in lactating women with past high lead exposure and low dietary calcium intake (Hernandez-Avila et al. 2003). The benefits of breastfeeding outweigh concern for lead at BLLs common among U.S. women (Lawrence 1997).

#### Drinking water

Lead is unlikely in source water but contaminates tap water through the corrosion of plumbing materials containing lead (Chin and Karalekas 1985; Levin 1986). Lead pipes are more likely to be found in older homes. In new homes, legally “lead-free” plumbing components can contain up to 8% lead (Safe Drinking Water Act Amendments of 1986). New plumbing leaches lead more readily than older fixtures, where mineral scale covers internal surfaces. The largest unaddressed sources of lead in water are brass or chrome-plated fixtures and illegal use of lead solder (U.S. EPA 2006b).

Cases of pediatric lead poisoning have been associated with drinking water (CDC 1994; Cosgrove et al. 1989; Shannon and Graef 1989). BLLs correlate with drinking water lead levels even in populations with low exposures (Lanphear et al. 1998b). Sampling drinking water to determine exposure is difficult, and it is easy for sporadic or short-term elevations to go undetected (Schock 1999). Hence, exposure to lead from drinking water may be underestimated (Testud et al. 2001).

Changing or introducing secondary disinfection practices (to kill waterborne pathogens) can affect lead levels in drinking water. After Washington, DC, switched disinfection agents, children in homes with lead service lines did not experience the almost 70% decrease in BLLs > 5 μg/dL experienced by other children (CDC 2004c). Children with lead service lines also had considerably higher BLLs (32% >5 μg/dL vs. 23% citywide) (CDC 2004c). Another study of changing disinfectants found that both water lead and BLLs increased (Miranda et al. 2007).

Lead levels in school drinking water can rise because long periods of nonuse (overnight, weekends, vacation) are followed by heavy consumption (Bryan 2004). The U.S. EPA has developed guidelines to help schools manage lead in their drinking water (U.S. EPA 2006d).

Drinking water contributes an estimated 10–20% of the total lead exposure of the general population (U.S. EPA 1991); formula-fed infants can have higher exposures. Drinking-water lead levels > 15 ppb are associated with a 14% increase in the percentage of children with BLLs > 10 μg/dL (Lanphear et al. 1998b).

#### Chocolate

Lead levels in chocolate products exceed those in other foods. In 1980, the market basket Total Diet Study (TDS) by the FDA found lead levels in chocolate milk more than three times those in whole milk, and levels in milk chocolate candy approximated those in canned foods (Pennington 1983). In the 2004 TDS, chocolate bars had the highest lead levels of the 280 items surveyed (FDA 2006a). A 2005 study comparing lead concentrations and isotopic compositions of cocoa beans grown in Nigeria with finished candy products found levels 60 times higher in finished candy versus cocoa beans (Rankin et al. 2005). No single source of lead was identified; levels rose at each stage of production.

#### Candy

Candy imported from Mexico is found repeatedly with high lead levels. Both candy and wrappers printed with lead ink have been cited (CDC 2002a; FDA 1995; Lynch et al. 2000; North Dakota Department of Health 2004). Lead-contaminated candy has also been imported from the Philippines and from Asian and Latin American countries. EBL cases have been reported in California, New York, North Dakota, Oklahoma, and Texas. In California, in 2001, candy was identified as a possible lead source for > 150 children with EBLs. In November 2006, the FDA reduced its recommended maximum lead level for candy consumed by children from 0.5 ppm to 0.1 ppm (FDA 2006b).

#### Imported foods

Foods and packaging produced outside the United States can contain high lead levels. Several spices (Sattar et al. 1989; Woolf and Woolf 2005), especially Hungarian paprika, have been contaminated (Kakosy et al. 1996). Food coloring also has been implicated in children’s EBLs (Vassilev et al. 2005). In 2006, California sued PepsiCo and Coca-Cola Co. concerning lead in the labels of bottles brought to the United States from Mexico (Lifsher 2006).

#### Dietary supplements

An assessment of 84 dietary supplements found lead in all, with 11 samples exceeding the tolerable dietary lead intake level (Dolan et al. 2003). These results correlate with other FDA data (Hight et al. 1993; Wong et al. 2004). Other herbal supplements associated with high levels of lead include nettle (FDA 2002) and supplements to treat hair loss (Health Canada 2004).

The Dietary Supplement Health and Education Act prevents the FDA from requiring premarket safety approval for supplements; hence, they require neither proof of safety nor efficacy (Marcus and Grollman 2002). The FDA recently proposed good manufacturing practice regulations to help ensure the safety of dietary supplements (FDA 2003b) and is developing a final rule.

#### Glass and dishes

Leaded crystal contains 24–32% lead oxide. Crystal decanters and glasses can release high amounts of lead in a short time, especially with cola (Guadagnino et al. 2000). The FDA has cautioned that children and pregnant women should avoid frequent use of crystal glassware and should not use lead crystal baby bottles (Farley 1998).

Ceramic pottery and other dinnerware containing lead glazes can be important exposure sources. Numerous reports of EBLs associated with homemade or low-fired ceramics from Mexico, southern Europe, North Africa, and the Middle East exist (Hellstrom-Lindberg et al. 2006; Manor and Freundlich 1983; Matte et al. 1994). Relatively new, commercially manufactured ceramic dinnerware has also been cited (CDC 2004a). The FDA has established criteria for leachable lead in ceramics ranging from 0.5 to 3.0 μg/mL, depending on the product (FDA 2005c).

Glassware with decals or painted surfaces can also contain lead (Sheets 1999). In 1979, the FDA and the U.S. glassware industry established a voluntary quality control program for decorated glasses that contain lead (FDA 1992). Since 1994, the FDA has exempted ornamental ceramicware from lead-leaching requirements if it contains a permanent marking warning “for decorative use only” (FDA 1992). A complete listing of dishware restricted for importation is available (FDA 2007b).

#### Vinyl lunchboxes

The U.S. FDA advised manufacturers and suppliers that lead in soft vinyl lunchboxes (FDA 2006c) may transfer to food. Thus, it could be deemed an unsafe food additive (under Section 409 of the Federal Food Drug and Cosmetic Act) (FDA 2008) and adulterated within the meaning of Section 402(a)(2)(C) of the statute and subject to regulation.

### Lead in consumer goods

According to the Consumer Product Safety Commission (CPSC), lead is the most frequently recalled substance that could result in poisoning. Many products associated with childhood lead poisoning are imported and do not meet U.S. standards (CDC 2002a; Geltman et al. 2001). A listing of all CPSC-recalled items is available (CPSC 2007). Products containing wood, metal, plastic, ceramics, and paper have been found with high lead concentrations.

#### Children’s products

Consumer goods with high lead content are found regularly. One study showed that 94% of plastic bread bags contained lead in the printing ink; a survey of families found that 16% reused bags to package children’s lunches (Weisel et al. 1991). In March and April 2007, CPSC issued recalls of 2,500 children’s painting easels, 128,700 toy sets, 400,000 key chains, 58,000 children’s necklaces, and 4 million children’s bracelets because of lead content. In August and September 2007, Mattel Inc. alone recalled 2.8 million lead-contaminated toys (Denver Post 2007). All of these items were made in China.

A study of toy jewelry found lead concentrations ≥ 50% in 40% of samples (Maas et al. 2005); when wiped, 70% of these samples released at least 1.0 μg lead, enough to cause high exposure with little handling. The scope and frequency of the recalls suggest that the current nonregulatory approach to controlling lead in children’s products could be strengthened.

#### Polyvinyl chloride (PVC)

Lead salts are used to stabilize polymers to avoid degradation from heat, sunlight, and wear. Although several studies demonstrate that dangerous lead exposures can occur with normal use of PVC products after extended use or exposure to sunlight, initial evaluation by CPSC found that lead in PVC products posed few risks to children (CPSC 1997).

An investigation of vinyl miniblinds found that they contaminate house dust and contribute significantly to lead toxicity in children (Norman et al. 1997; West et al. 1998). Because about 30 million sets are sold annually and the polymers degrade under normal conditions, this might be a lead exposure source for millions of children, particularly those living in manufactured housing commonly equipped with miniblinds.

Since 1977, the water pipe market has more than doubled, and 80% of new drinking water and wastewater pipes are plastic, mostly PVC (Vinyl News Service 2006). Early tests of PVC pipes showed that lead contamination could be high (National Academy of Sciences Safe Drinking Water Committee 1982). Despite a standardized testing procedure for plastic pipes to reduce the potential for high lead exposures [Mitchener 1992; NSF/ANSI (American National Standards Institute) 2008; U.S. EPA 2007e], reports of dangerous exposures from plastic pipes continue (Koh et al. 1991).

Artificial Christmas trees made of PVC also degrade under normal conditions (Maas et al. 2004). About 50 million U.S. households have artificial Christmas trees, of which about 20 million are at least 9 years old, the point at which dangerous lead exposures can occur. High lead levels have also been found in telephone cords (Abdul-Razzaq et al. 2003).

#### Synthetic turf

Synthetic turf is currently used on about 3,500 playing fields throughout the United States (Claudio 2008). Rubber infill or crumbs made from recycled tires keep the turf blades upright, and this rubber can contain lead. The exposure potential, especially on older fields that have accumulated dust and where the materials are deteriorating, is a research gap.

#### Candle wicks

Candles with a lead metal core contribute to lead in the home (Nriagu and Kim 2000; van Alphen 1999). Exposure occurs both from air and from hand-to-mouth activity. However, to date, no children’s EBLs traceable to candles have been reported. In 2002, the CPSC banned candlewicks containing > 0.06% lead (CPSC 2003).

### Lead paint in housing

Approximately 38 million homes had lead-based paint (LBP) in 2000 (Jacobs and Nevin 2006). Of those, an estimated 24 million units had deteriorated lead paint, dust lead, or bare soil contaminated with lead (Jacobs et al. 2002). Of those with LBP hazards, 1.2 million units housed low-income families with children < 6 years of age. A relatively small number of properties may account for large numbers of children with EBLs (Korfmacher and Kuholski 2007; Meyer et al. 2005; Reyes et al. 2006).

Housing units with LBP hazards are not evenly distributed (Jacobs et al. 2002). In 2000, for households with incomes ≤ $30,000—the federal poverty level at that time—35% of the housing units had LBP hazards compared with 19% of all housing units. Northeast and Midwest housing has twice the prevalence of LBP hazards compared with housing in the South and West. Although the prevalence of LBP hazards increases with the age of the building, most painted surfaces, even in older housing, do not have lead paint; only 2–25% of building components have LBP (Jacobs et al. 2002).

Children in units with LBP are almost 10 times more likely to have an EBL than children in similar housing without lead paint (Schwartz and Levin 1991). Addressing lead paint hazards significantly reduces the risk of identifying another child with an EBL in a unit where one was previously identified (Brown et al. 2001a).

Mean BLLs of children whose housing was abated show a 38% decrease over a 2-year period after lead hazard control (National Center for Healthy Housing and the University of Cincinnati Department of Environmental Health 2004). Nonetheless, disturbing lead painted surfaces can increase the BLLs of children living in those units during repair work unless appropriate controls are instituted, especially dust clearance levels (Amitai et al. 1991; Bellinger et al. 1986; HUD 1995). Studies of well-conducted renovation activities show that although lead hazard interventions reduce most children’s BLLs, about 10% of the time BLLs significantly increased (CDC 1997; Clark et al. 2004); young children (< 18 months of age) are at highest risk of increases. BLLs of children who continued to live in the house or relocated for less than the full work period also were significantly more likely to increase than those of children who relocated for the entire renovation. Consequently, remediation and abatement activities that disturb lead paint must be followed by specialized cleaning and dust-lead testing to determine whether the unit is safe for re-occupancy.

## Risk Factors for EBLs in U.S. Children

Between 1976 and 2002, the National Health and Nutrition Examination Surveys (NHANES) identified a constellation of risk factors for EBLs among children. Previously undocumented risk factors continue to be uncovered in urban areas and within particular subpopulations (Dignam et al. 2004). Nationally representative samples do not identify or characterize local risks. The CDC recommends that states target communities with the highest risk for lead exposure, using established risk factors (CDC 2003).

### Age

Children’s BLLs peak around 15–24 months of age (Tong et al. 1996). This age dependence persists even as average BLLs have decreased. Given the pervasive lead contamination of our environment, it is not surprising that normal hand-to-mouth behaviors result in high exposures among toddlers. Young children also absorb lead more readily than do older children and adults. Exposures with little effect on adults cause high levels in young children (Faustman et al. 2000).

### Race and ethnicity

**Figure 3:**
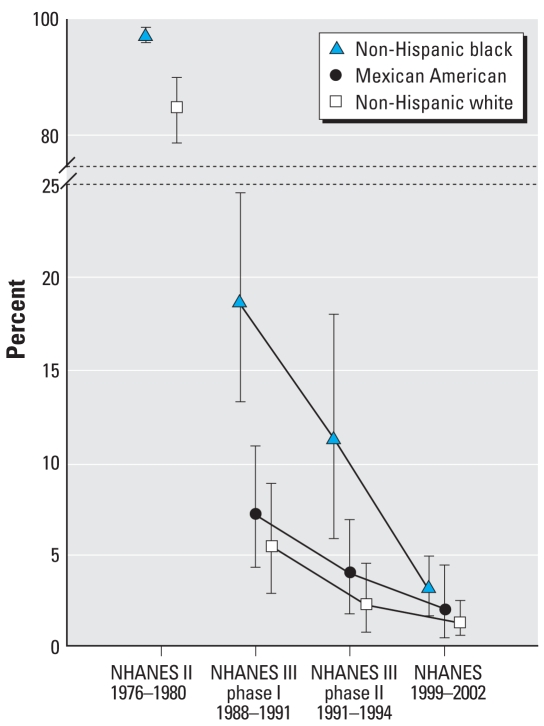
Percentage of U.S. children, 1–5 years of age, with EBLs ≥10 μg/dL (95% confidence intervals), by race/ethnicity. Data from CDC (2005b).

The NHANES show an association between BLLs and race/ethnicity (Figure 3). In 1976–1980, the geometric mean BLL for all U.S. children was 16 μg/dL versus 21 μg/dL for black children (Mahaffey et al. 1982). Data from 1999–2002 show similar patterns: 46.8% of non-Hispanic black children and 27.9% of Mexican-American children exceeded 5 μg/dL compared with 18.7% for white children (CDC 2005b). Fortunately, the gap is narrowing. The most recent national data show that non-Hispanic black children had the largest decline in BLLs (72%) of all racial and ethnic groups, reducing the differences between subpopulations (Jones R, personal communication).

#### Use of ethnic remedies, cosmetics, and goods

Folk medicines and remedies from many cultures can contain high lead levels (Baer and Ackerman 1988; Trotter 1985). Traditional Mexican remedies were the earliest focus (CDC 2002a), but poisonings in six states and one death have been linked to Ayurveda, a traditional South Asian medicine (CDC 1984, 2004d; Moore and Adler 2000). Imported herbal remedies are available at many local markets (Saper et al. 2004). Ethnic and imported cosmetics and other goods have also been associated with high lead exposures (CDC 2005c; Sprinkle 1995).

#### Immigrant or refugee status

Refugee, internationally adopted, and recent immigrant children are more likely than U.S.-born children to have EBLs, both on arrival in the country and later (Geltman et al. 2001; Miller and Hendrie 2000; Tehranifar et al. 2008). Many foreign children enter the United States with EBLs resulting from lead sources in their native countries. Their BLLs rise after resettlement because of both lead contamination in their new environments and continued use of imported products containing lead. Existing health burdens and cultural, language, and economic barriers compound the risk for lead poisoning after resettlement. For example, iron deficiency, prevalent among refugee children, increases lead absorption through the gastrointestinal tract. Exposure to small amounts of lead can result in very high BLLs in iron-deficient children (Stauffer et al. 2002; Weissman 1994).

An increased risk for EBLs has been documented among refugee and immigrant children from Africa, Cuba, China, Russia, Thailand, and other countries (CDC 2005a; Mielke et al. 1984; Trepka et al. 2005). For instance, although there were only 46 cases of EBLs in Manchester, New Hampshire, in 1997, there were 88 in 2004; all the additional EBLs were among African-born children. In 2003, the CDC found that 45% of refugee children had elevated BLLs a few months after resettlement (CDC 2005a). BLLs are often elevated in school-age and teenage foreign-born children. The CDC recommends testing refugee and immigrant children on entry to the United States and again 3–6 months later, mirroring policies established by New Hampshire’s CLPPPs after a fatality in 2000. The CDC also recommends nutritional evaluation and intervention for deficiencies.

### Income level

Children with EBLs are more common in communities with many households below the federal poverty level, independent of housing age or proportion of black children (Bernard and McGeehin 2003; Sargent et al. 1995). In 1976–1980, children with the lowest family income had an average BLL of 20 μg/dL versus 16 μg/dL nationally (Mahaffey et al. 1982). In Massachusetts in 1991–1992, the 15 communities with > 25% of children ≤ 5 years old living in poverty accounted for 71% of children with BLLs ≥ 25 μg/dL (Sargent et al. 1995).

Income-based disparities of EBLs in children have narrowed. In 1991–1994, the percent of children with EBLs was 4.5% in the lowest income group versus 0.7% in the highest income group (Pirkle et al. 1994). By 1999–2002, the difference between the percent of Medicaid-enrolled children with EBLs and the general population was not statistically significant (1.7% vs. 1.3%, respectively). However, the geometric mean BLL for Medicaid-enrolled children exceeds unenrolled children, indicating continued disparity in lead exposures (2.6 μg/dL vs. 1.7 μg/dL) (CDC, unpublished data).

### Age of housing

Housing built before the 1978 ban on lead paint is a significant risk factor for exposure. Forty-two percent of children living in housing built before 1946, and 39% of children in housing built between 1946 and 1973 had BLLs ≥5 μg/dL versus 14% of children in housing built after 1973 (Bernard and McGeehin 2003).

### Location of residence

Children 1–5 years of age living in the 10 largest U.S. cities accounted for 46% of EBLs reported to the CDC in 2003 but only 7% of the population that age (CDC, unpublished data). Usually, EBL cases are clustered within cities. A 2001 study of seven cities found that 50% of children with EBLs lived in 11% of the ZIP codes in those cities (Brown et al. 2001b).

Lead contamination typically is greater in urban versus rural areas (National Research Council 1993; U.S. EPA 2006a). Although long-distance transport of lead does occur, many studies show that most of the lead emitted in urban areas remains there (Flegal et al. 1989). The discrepancy between BLLs of urban and rural children has remained constant despite the decline in overall lead exposures for U.S. children since the late 1970s (Brody et al. 1994).

### Parental occupations

Lead dust from work inadvertently carried by parents settles on surfaces and workers’ clothing, where it can be ingested or inhaled by young children (Hipkins et al. 2004). Children of lead-exposed workers have disproportionately higher BLLs (Chan et al. 2000; Whelan et al. 1997). Based on 1981–1983 survey data, an estimated 48,000 families with children < 6 years of age had a household member who worked with lead (Roscoe et al. 1999). Concern for take-home exposure is not new; two studies from the early 1900s identified severe poisonings of workers’ families, including case histories from 1860 (Holt 1923; Oliver 1914).

Many occupations with potential high lead exposures are exempted from Occupational Safety and Health Administration workplace protections, including transportation workers, most public employees, and self-employed workers in industries such as battery reclamation, automobile repair, pottery and ceramics, and stained glass. Undocumented workers are particularly vulnerable because of limited access to exposure monitoring and protective measures.

### Other risk factors

#### Season of the year

BLLs are significantly higher in warm weather in both national and local studies (Kaufmann et al. 2000; U.S. EPA 2007c). The relation persists despite the decline in lead exposure. Several factors may explain seasonal variations: greater exposures to soil lead, dispersion of dust when lead-painted windows are opened and shut (Haley and Talbot 2004), and remobilization of lead on interior surfaces as air moves through open windows and doors. In warmer weather, children’s longer hours outdoors may increase exposure to airborne and soil lead and contribute to seasonality in BLLs (Yin et al. 2000). Changes in Vitamin D exposures during the warmer weather may also account for some of the seasonality observed (Kemp et al. 2007).

#### Tobacco smoke

Having a smoker in the house has been associated with higher BLLs in children for 30 years (Willers et al. 1988; Zielhuis et al. 1978). Cotinine levels still correlate positively with BLLs (Mannino et al. 2003).

## Implications for Lead Poisoning Prevention

The current CDC advisory level for intervention in individual children is 10 μg/dL (CDC 1991). It is not a safe level; studies show strong and long-lasting effects with BLLs as low as 2 μg/dL. Therefore, the CDC recommends primary prevention—that is, that all lead sources in children’s environments be controlled or eliminated before children are exposed.

Achieving the Healthy People 2010 objective—to reduce BLLs as much as possible and to eliminate childhood lead poisoning— will require collaboration by all levels of government. This cannot succeed without enforcing all existing standards, ensuring that ambient lead levels continue to decline, and reversing recent trends of increased lead exposures, such as air lead and imported consumer goods. Table 2 summarizes federal authorities for regulating lead.

### Addressing lead paint hazards

Lead-based paint in housing remains the most common high-dose source of lead in children’s environments. Reducing lead hazards in housing requires

- Data to be shared across organizational boundaries
- Local and state regulatory requirements for lead-safe housing
- Strengthened enforcement of existing laws, especially cleanup
- Greater public and private investment for lead hazard control.

Some of the most hazardous residential units may not be eligible for HUD’s Lead Hazard Control program because they are uninsured, have outstanding taxes, have other serious code violations, or because the owner cannot be located. In this case, emergency funds are needed to raze buildings that cannot reasonably be made safe. Evidence that primary prevention is effective is mounting. For example, a project initiated in 1998 by HUD, assisted by the Department of Justice, the CDC, and the U.S. EPA, to enforce Title 1018 of the Toxic Substances Control Act has resulted in commitments to make over 185,000 high-risk properties lead-safe by 2006 (Gant J, HUD, personal communication).

### Identifying all sources of lead exposure

Local CLPPPs remain the frontline in identifying lead exposure sources. As particular lead paint hazards are controlled or eliminated, other lead sources assume greater importance and visibility. The CDC recommends that when children with EBLs are identified, CLPPPs identify all sources of lead in the child’s environment (CDC 2002b). Research is needed on effective intervention strategies for children with BLLs above average but < 10 μg/dL to prevent dangerous exposures.

### Maintaining lead-safe communities

Creating lead-safe communities can occur only with the active involvement of all levels of government—local, state, and federal—and will depend on several strategies. Foremost are systems that monitor and evaluate all children’s potential lead exposures. Other keys to institutionalizing primary prevention are requirements for lead-safe housing and work practices, dust- and soil-lead testing after repairs in older housing, identification of all lead sources for children with EBLs, elimination of products with dangerous lead levels, and timely mechanisms to share information about lead sources, including toxic properties, across government agencies.

State and local officials should evaluate whether their existing primary prevention efforts sufficiently protect children.

Federal agencies should support local and state efforts by

- Monitoring lead in air, drinking water, food, and consumer products
- Enforcing laws that control lead contamination
- Educating specific populations about lead and controlling exposures
- Improving exposure modeling techniques, accounting for all sources of exposure
- Conducting research and ongoing evaluation of lead poisoning prevention activities.

## Conclusions

The Healthy People 2010 objective to eliminate BLLs ≥ 10 μg/dL is within our grasp. The course is clear. We must identify and address all existing lead hazards and be vigilant in preventing new hazards. Recent research describes the enormous societal benefits to be reaped from preventing lead exposure in children (Grosse et al. 2002; Landrigan et al. 2002; Nevin et al. 2008), with total annual estimates of $43–110 billion or more. The overall reduction of lead in the environment will benefit all U.S. children—and adults, too.

## Correction

In “Sources of Lead Exposure,” the percentages given for types of sources were incorrect in the manuscript originally published online. They have been corrected here.

## Figures and Tables

**Table 1:** Lead used in Massachusetts manufacturing, 2003.

Activity/facility type	No. of facilities	Total use (lb)
Municipal waste combustors	7	2,642,987
Wire and cable manufacturing	21	2,622,713
Rubber and plastics manufacturing	10	1,856,941
Hazardous waste facilities	1	714,118
Fabricated metals manufacturing	22	363,406
Chemicals and allied products	12	304,619
Primary metals manufacturing	8	157,742
Electronic equipment manufacturing	37	119,651
Others	134	503,451
Total	252	9,285,628

Data from Massachusetts Department of Environmental Protection (2005).

**Table 2:** U.S. lead regulatory authorities.

Agency	Lead source regulated	Statutory authority	Voluntary
CPSC	Paint/coatings	CPSC 1977	None
	Candle wicks	CPSC 2003	None
	Lead in products intended for use by children	None	CPSC 2008
FDA	Food/materials that contact food (domestic)	FDA 2004a	None
	Lead in bottled water	FDA 2003a	None
	Prescription and over-the-counter drugs	FDA 2004b	None
	Dietary supplements	Proposed rule (FDA 2003b)	None
	Seizure of imported food, drugs, and cosmetics	FDA 2003c	None
	Candy	None	FDA 2006a
	Ceramics/pottery	None	FDA 2005a
	Shellfish	None	FDA 2005b
	Wine	None	FDA 2007a
	Soft vinyl lunchboxes	None	FDA 2006b
U.S. EPA	Drinking water	U.S. EPA 1991	None
	Plumbing components, school drinking water	U.S. EPA 1988, 2007c	U.S. EPA 2008a
	Air	U.S. EPA 2008b	None
	Lead paint disclosure, renovation/repair, and clean up	U.S. EPA 1992, U.S. EPA 2006c	None
	Waste management, disposal	U.S. EPA 1980a, U.S. EPA 1980b	None
HUD	Residential lead paint hazards in federally subsidized properties	HUD 1999	None
	Disclosure of lead paint at property transfer	HUD 1992	None
OSHA	Worker protection for general industry	OSHA 2008a	None
	Construction industry	OSHA 2008b	None
NSF/ANSI	Plumbing codes, plumbing components	Local and state housing and plumbing codes	NSF/ANSI 2008
			U.S. EPA 2007e
